# A note on a generalized double series

**DOI:** 10.1371/journal.pone.0340358

**Published:** 2026-01-16

**Authors:** Robert Reynolds

**Affiliations:** Department of Mathematics and Statistics, York University, Toronto, Ontario, Canada; University of Education, PAKISTAN

## Abstract

By employing contour integration the derivation of a generalized double finite series involving the Hurwitz-Lerch zeta function is used to derive closed form formulae in terms of special functions. We use this procedure to find special cases of the summation and product formulae in terms of the Hurwitz-Lerch zeta function, trigonometric functions and the gamma function. A short table of quotient gamma functions and plots are produced for easy reading by interested readers.

## Theoretical background and applications

The double series expression in this work is connected to several areas of mathematics, particularly special functions, complex analysis, and analytic number theory. The main component of the double series is the Hurwitz-Lerch function, Φ(z,s,a). This special function is widely used in analytic number theory [DLMF, 25.16] and mathematical physics, generalizing the Riemann [DLMF, 25.11.2] and Hurwitz zeta functions [DLMF, 25.14.2], and the polylogarithm, [DLMF, 25.17]. The series also contains terms like $e^{im(\ldots )}$ which has ties to the theory of exponential sums [1–3]. These sums are used in analytic number theory for studying the distribution of various sequences, including prime numbers [DLMF, 25.16]. The structure of the summation, particularly the alternating signs and the nested form, involves a series resummation and a known identity featuring special functions. Sometimes, these types of expressions can be derived from integral representations or generating functions. While the expression is purely mathematical, functions like the Hurwitz-Lerch function and complex exponential sums find applications in theoretical physics, including quantum field theory, statistical mechanics, and condensed matter physics [DLMF, 25.17].

The double series in this work is not a double Fourier series. see chapter XVII in [4], but shares some slight similarities while being different. Its components, such as the complex exponentials and double summation, come from similar mathematical backgrounds but are organized to study number-theoretic functions rather than to represent a two-dimensional periodic function. The summand terms in Eq (13) involves the Hurwitz-Lerch zeta function, which itself is an infinite series, while the summand in a double Fourier series uses elementary basis functions involving products of sines and cosines, or complex exponentials namely $e^{i\left(\frac{2\pi mx}{L_{1}}\;+\;\frac{2\pi ny}{L_{2}}\right)}$. In Eq (13), the indices *p* and *j* run over a finite, specific range, defined by the parameters *n* and *z*, while in a double Fourier series the indices are over the real line or the positive half real line. A double Fourier series is a periodic function of two variables decomposed into its fundamental and frequency components. The terms in Eq (13) are not necessarily orthogonal, but the basis functions in a double Fourier series are over the domain of the function. The double Fourier series can be thought of as a useful tool for breaking down periodic functions. A double summation and complex exponentials are used in Eq (13), which is a specialized and non-periodic expression.

In the work by Qureshi et al. [5], the authors derive identities for double-series using hypergeometric functions, which can then be used to derive transformation formulas between different types of hypergeometric functions. In the work by Dinesh et al. [6], the authors evaluated finite double summation relations for the multivariable *A*-function using the summation of a double hypergeometric series. In the work by Andrews et al. [7], the authors proved new double summation hypergeometric *q*-series representations for several families of partitions, including those that appear in the famous product identities of Göllnitz, Gordon, and Schur. Several different proofs for their results, using bijective partitions mappings and modular diagrams, the theory of *q*-difference equations and recurrences, and the theories of summation and transformation for *q* -series were given. General family of similar double series and a number of other interesting special cases were highlighted.

## Introduction

Generalized double series and their extensions to multiple series are important tools in contemporary applied analysis, appearing in the solution of partial differential equations, special functions of several variables, lattice sums, asymptotic expansions, and modern spectral methods for fractional-order problems. Recent work has been conducted in classical theory of convergence and smoothness for double Fourier series and in the development of efficient computational frameworks that produce rapidly convergent double or multiple series representations for complex multidimensional problems. Some notable works are by [8–13], Research in the applied analysis are listed in the works by; Khalil et al. [14], where the authors “studied shifted Jacobi polynomials in the case of two variables and develop some new operational matrices of fractional-order integrations as well as fractional-order differentiations”, in the work by Tailb et al. [15], the authors proposed the numerically stable operational matrices to approximate the Caputo fractional-order derivatives by introducing an algorithm. In the work by Khalil et al. [16], the authors extended the operational matrices technique to design a spectral solution of nonlinear fractional differential equations (FDEs). The work by Khalil et al. [17], was to find the approximate solution of a general FPDEs subject to nonlocal integral type boundary conditions on both ends of the domain. The work by Nazir et al. [18], was to present an analytical solution for measles spread model with three doses of vaccination using Caputo–Fabrizio fractional derivative (CFFD). The work by Abuteen et al. [19] proposes an analytical-numerical approach for providing solutions of a class of nonlinear fractional Klein-Gordon equation subjected to appropriate initial conditions in Caputo sense by using the Fractional Reduced Differential Transform Method (FRDTM). The work by Qiu et al. [20], systematically investigates the optimal harvesting of a stochastic delay competitive Lotka–Volterra model with Lévy jumps. The work by Tadoummant et al. [21], was to investigate the solution of fractional-order partial differential equations and their coupled systems. In the work by Khalil et al. [22], extended the operational matrix method for approximating the solution of the fractional-order two-dimensional elliptic partial differential equations (FPDEs) under nonlocal boundary conditions. The work by Hammad et al. [23], was to investigate the atomic solution of a special type of fractional partial differential equations. The combination of convergence theory for classical double series with new series-based numerical schemes, studied by current researchers expands the range of problems in heat transfer, electrostatics, epidemiology, and fractional calculus that can be treated analytically and numerically with high precision. Finite sums of special functions are recorded in chapter 5 in [24]. Eqs (5.3.10.1) and (5.3.10.2) in [25] yield finite summations of the Hurwitz-zeta function Φ(z,s,a) in terms of the Hurwitz-zeta function. In [26] an infinite sum involving the Hurwitz-Lerch zeta function was studied. The underlying formula transformed by contour integration is the infinite series involving the tangent function whose angle is a multiple of 2 raised to an integer power. The work done in [26] was centred around evaluating an infinite sum in terms of special functions, while in the current paper the author studies double finite series of special functions. The work by Kaluszka et al. [27], presents stochastic analogs of classical formulas for the gamma function, representing some random variables as finite or infinite products of independent random variables. The work by Krysicki, [28], explores the properties of the beta prime distribution in the context of random variable decomposition. The paper’s main focus is to show how a random variable with a beta prime distribution can be expressed as a finite or infinite product of independent random variables.

In the current paper, the underlying function transformed by contour integration has a more generalized angle form which allows for more interesting evaluations in terms of trigonometric and special functions. The reason behind this research, is to expand on literature involving the finite double sum and the product of special functions, such as the gamma function. The new results found in this work, lies in the derivation and evaluation involving the double finite product of quotient gamma functions which is not present in current literature to the best of my knowledge. We use a contour integral method and apply it to a generalized double finite summation formula involving the secant function to study the resulting double finite summation of the Hurwitz-Lerch zeta function. Since the Hurwitz-Lerch zeta function is a special function it has several composite functions which can yield new results by algebraic means. Some of these composite functions are the gamma function and other trigonometric functions. The objectives pursued in this work were the derivation of a double finite sum involving the Hurwitz-Lerch zeta function, along with double finite products involving the gamma function and a few functional identities for the Hurwitz-Lerch zeta function. In this work we apply the contour integral method [29], to the the generalized form of the finite secant sum on page 90 in [30] to derive a generalized form involving the finite sum of the secant function, resulting in


$$\frac{1}{2\pi i}\int_{C}\sum_{p=0}^{n}a^{w}w^{-1-k}(\left(1+(-1{)}^{1+z}\right)\sec\left((m+w)(1+2z{)}^{p}\right)$$

$$+\sum_{j=1}^{z}2(-1{)}^{1+j+z}\cos\left(2j(m+w)(1+2z{)}^{-1+p}\right)$$

$$\;\sec\left((m+w)(1+2z{)}^{p}\right))dw$$

$$=\frac{1}{2\pi i}\int_{C}a^{w}w^{-k-1}\left(\sec\left((m+w)(2z+1{)}^{n}\right)-\sec\left(\frac{m+w}{2z+1}\right)\right)dw$$ (1)

where $a,m,k\in \mathbb{C},Re(m\;+\;w)>0,n\in Z^{+},z\in Z^{+}$. Using Eq (1) the main Theorem to be derived and evaluated is given by


$$\sum_{p=0}^{n}\left((-1{)}^{z+1}+1\right){\left((2z+1{)}^{p}\right)}^{k}e^{im(2z+1{)}^{p}}\Phi \left(-e^{2im(2z+1{)}^{p}},-k,\frac{1}{2}\left(a(2z+1{)}^{-p}+1\right)\right)$$

$$+\sum_{p=0}^{n}\sum_{j=1}^{z}(-1{)}^{j+z+1}{\left((2z+1{)}^{p}\right)}^{k}e^{im(-2j+2z+1)(2z+1{)}^{p-1}}$$

$$(\Phi \left(-e^{2im(2z+1{)}^{p}},-k,\frac{1}{2}\left(a(2z+1{)}^{-p}+1-\frac{2j}{2z+1}\right)\right)$$

$$+e^{4ijm(2z+1{)}^{p-1}}\Phi \left(-e^{2im(2z+1{)}^{p}},-k,\frac{j}{2z+1}+\frac{1}{2}\left(a(2z+1{)}^{-p}+1\right)\right))$$

$$={\left((2z+1{)}^{n}\right)}^{k}e^{im(2z+1{)}^{n}}\Phi \left(-e^{2im(2z+1{)}^{n}},-k,\frac{1}{2}\left(a(2z+1{)}^{-n}+1\right)\right)$$

$$-{\left(\frac{1}{2z+1}\right)}^{k}e^{\frac{im}{2z+1}}\Phi \left(-e^{\frac{2im}{2z+1}},-k,\frac{1}{2}(2za+a+1)\right)$$ (2)

where the variables *k*,*a*,*m* are general complex numbers and *n*,*z* are positive integers. The derivations follow the method used by us in [29]. This method involves using a form of the generalized Cauchy’s integral formula given by

$$\frac{y^{k}}{\Gamma (k+1)}=\frac{1}{2\pi i}\int_{C}\frac{e^{wy}}{w^{k+1}}dw,$$ (3)

where y,w∈ℂ and *C* is in general an open contour in the complex plane where the bilinear concomitant [29] is equal to zero at the end points of the contour. This method involves using a form of Eq (3) then multiplies both sides by a function, then takes the double finite sum of both sides. This yields a double finite sum in terms of a contour integral. Then we multiply both sides of Eq (3) by another function and take the infinite sum of both sides such that the contour integral of both equations are the same.

## The Hurwitz-Lerch zeta function

We use Eq (1.11.3) in [31] where Φ(z,s,v) is the Hurwitz-Lerch zeta function which is a generalization of the Hurwitz zeta ζ(s,v) and Polylogarithm functions *Li*_*n*_(*z*). In number theory and complex analysis, the Hurwitz-Lerch zeta function is a mathematical function that appears in many branches of mathematics and physics. It is named after Czech mathematician Mathias Lerch, who published a paper about the function in 1887. Numerous areas of mathematics, including number theory (especially in the investigation of the Riemann zeta function and its generalizations), complex analysis, and theoretical physics, all have uses for it. It can be used to express a variety of complex functions and series and is involved in numerous mathematical identities. The Hurwitz-Lerch zeta function has a series representation given by

$$\Phi (z,s,v)=\sum_{n=0}^{\infty }(v+n{)}^{-s}z^{n}$$ (4)

where |z|<1,v≠0,−1,−2,−3,.., and is continued analytically by its integral representation given by

$$\Phi (z,s,v)=\frac{1}{\Gamma (s)}\int_{0}^{\infty }\frac{t^{s-1}e^{-(v-1)t}}{e^{t}-z}dt$$ (5)

where *Re*(*v*) > 0, and either |z|≤1,z≠1,Re(s)>0, or z=1,Re(s)>1.

## 1 Contour integral representation for the finite sum of the Hurwitz-Lerch zeta functions

In this section we derive the contour integral representations of the left-hand side and right-hand side of Eq (1) in terms of the Hurwtiz-Lerch zeta and trigonometric functions. A contour integral representation is a technique in complex analysis that expresses a function as an integral of another function along a specific path or contour in the complex plane. This approach provides a useful tool for extending a function’s domain of definition, proving identities, and evaluating sums.

### 1.1 Derivation of the generalized Hurwitz-Lerch zeta contour integral for the secant function

We use the method in [29]. Using Eq (3) we first replace *y* by log(a)+ib(2y+1) and multiply both sides by $-2i(-1{)}^{y}e^{ibm(2y+1)}$ then take the infinite sum over y∈[0,∞) and simplify in terms of the Hurwitz-Lerch zeta function to get


$$-\frac{i2^{k+1}(ib{)}^{k}e^{ibm}\Phi \left(-e^{2ibm},-k,\frac{b-i\log(a)}{2b}\right)}{\Gamma (k+1)}$$

$$\;=\frac{1}{2\pi i}\sum_{y=0}^{\infty }\int_{C}(-1{)}^{y}a^{w}w^{-k-1}e^{ib(2y+1)(m+w)}dw$$

$$\;=\frac{1}{2\pi i}\int_{C}\sum_{y=0}^{\infty }(-1{)}^{y}a^{w}w^{-k-1}e^{ib(2y+1)(m+w)}dw$$

$$\;\;=-\frac{1}{2\pi i}\int_{C}ia^{w}w^{-k-1}\sec(b(m+w))dw$$ (6)

from Eq (1.232.2) in [32] where Re(w+m)>0 and Im(m+w)>0 in order for the sums to converge. We apply Tonelli’s theorem for multiple sums, see page 177 in [33] as the summands are of bounded measure over the space ℂ×[0,∞).

### 1.2 Derivation of a generalized Hurwitz-Lerch zeta function in terms of the product of the cosine and secant function contour integral

We use the method in [29]. Using a generalization of Cauchy’s integral formula (3) we first replace *y* by log(a)+ix+y then multiply both sides by *e^mxi^* then form a second equation by replacing *x* by –*x* and add both equations to get


$$\frac{e^{-imx}\left(e^{2imx}(\log(a)+ix+y{)}^{k}+(\log(a)-ix+y{)}^{k}\right)}{\Gamma (k+1)}$$

$$\;=\frac{1}{2\pi i}\int_{C}2w^{-k-1}e^{w(\log(a)+y)}\cos(x(m+w))dw$$ (7)

Next we replace *y* by ib(2y+1) and multiply both sides by $(-1{)}^{y}e^{ibm(2y\;+\;1)}$ and take the infinite sum over y∈[0,∞) and simplify in terms of the Hurwitz-Lerch zeta function to get


$$\frac{2^{k}(ib{)}^{k}e^{im(b-x)}\left(\Phi \left(-e^{2ibm},-k,\frac{b-x-i\log(a)}{2b}\right)+e^{2imx}\Phi \left(-e^{2ibm},-k,\frac{b+x-i\log(a)}{2b}\right)\right)}{\Gamma (k+1)}$$

$$=\frac{1}{2\pi i}\sum_{y=0}^{\infty }\int_{C}2(-1{)}^{y}a^{w}w^{-k-1}e^{ib(2y+1)(m+w)}\cos(x(m+w))dw$$

$$=\frac{1}{2\pi i}\int_{C}\sum_{y=0}^{\infty }2(-1{)}^{y}a^{w}w^{-k-1}e^{ib(2y+1)(m+w)}\cos(x(m+w))dw$$

$$=\frac{1}{2\pi i}\int_{C}a^{w}w^{-k-1}\sec(b(m+w))\cos(x(m+w))dw$$ (8)

from Eqs (1.232.2) and (1.411.3) in [32] where Re(w+m)>0 and Im(m+w)>0 in order for the sums to converge. We apply Tonelli’s theorem for sums and integrals, see page 177 in [33] as the summand and integral are of bounded measure over the space ℂ×[0,∞).

### 1.3 Derivation of the contour integrals

In this section we will use Eqs (6) and (8) and simple substitutions to derive the contour integrals in Eq (1).

#### 1.3.1 Left-hand side first contour integral.

Use Eq (6) and replace *b* by $(2z\;+\;1{)}^{p}$ then multiply both sides by $(-1{)}^{z+1}\;+\;1$ and take the finite sum over p∈[0,n] to get;


$$\sum_{p=0}^{n}\frac{1}{k!}2^{k+1}\left((-1{)}^{z+1}+1\right){\left(i(2z+1{)}^{p}\right)}^{k}e^{im(2z+1{)}^{p}}$$

$$\Phi \left(-e^{2im(2z+1{)}^{p}},-k,\frac{1}{2}(2z+1{)}^{-p}\left((2z+1{)}^{p}-i\log(a)\right)\right)$$

$$\;=\frac{1}{2\pi i}\int_{C}\sum_{p=0}^{n}\left((-1{)}^{z+1}+1\right)a^{w}w^{-k-1}\sec\left((m+w)(2z+1{)}^{p}\right)dw$$ (9)

#### 1.3.2 Left-hand side second contour integral.

Use Eq (8) and replace *x* by $2j(2z\;+\;1{)}^{p-1}$, and *b* by $(2z\;+\;1{)}^{p}$ then multiply both sides by 2(−1)^*j* + *z* + 1^ and take the finite sums over p∈[0,n] and j∈[1,z] to get;


$$\sum_{p=0}^{n}\frac{1}{k!}2^{k+1}(-1{)}^{j+z+1}{\left(i(2z+1{)}^{p}\right)}^{k}\exp\left(im\left((2z+1{)}^{p}-2j(2z+1{)}^{p-1}\right)\right)$$

$$\sum_{j=1}^{z}(\Phi \left(-e^{2im(2z+1{)}^{p}},-k,\frac{1}{2}(2z+1{)}^{-p}\left(-2j(2z+1{)}^{p-1}+(2z+1{)}^{p}-i\log(a)\right)\right)$$

$$+e^{4ijm(2z+1{)}^{p-1}}\Phi \left(-e^{2im(2z+1{)}^{p}},-k,\frac{\left(2j(2z+1{)}^{p-1}+(2z+1{)}^{p}-i\log(a)\right)}{2(2z+1{)}^{p}}\right))$$

$$=\frac{1}{2\pi i}\int_{C}\sum_{p=0}^{n}\sum_{j=1}^{z}2a^{w}(-1{)}^{j+z+1}\frac{\cos\left(2j(m+w)(2z+1{)}^{p-1}\right)}{\cos\left((m+w)(2z+1{)}^{p}\right)}\frac{dw}{w^{k+1}}$$ (10)

#### 1.3.3 Right-hand side first contour integral.

Use Eq (6) and replace *b* by $\frac{1}{2z\;+\;1}$ then multiply both sides by –1 to get;


$$-\frac{1}{k!}2^{k+1}{\left(\frac{i}{2z\;+\;1}\right)}^{k}e^{\frac{im}{2z+1}}\Phi \left(-e^{\frac{2im}{2z+1}},-k,\frac{1}{2}(2z+1)\left(\frac{1}{2z+1}-i\log(a)\right)\right)$$

$$=-\frac{1}{2\pi i}\int_{C}a^{w}w^{-k-1}\sec\left(\frac{m+w}{2z+1}\right)dw$$ (11)

#### 1.3.4 Right-hand side second contour integral.

Use Eq (6) and replace *b* by (2*z* + 1)^*n*^ to get;


$$\frac{1}{k!}2^{k+1}{\left(i(2z+1{)}^{n}\right)}^{k}e^{im(2z+1{)}^{n}}\Phi \left(-e^{2im(2z+1{)}^{n}},-k,\frac{\left((2z+1{)}^{n}-i\log(a)\right)}{2(2z+1{)}^{n}}\right)$$

$$=\frac{1}{2\pi i}\int_{C}a^{w}w^{-k-1}\sec\left((m+w)(2z+1{)}^{n}\right)dw$$ (12)

## 2 Finite sum of the Hurwitz-Lerch zeta function: evaluations and interpretations of results

In this section we will derive and evaluate formulae involving the finite double sum of the Hurwitz-Lerch zeta function in terms other special functions, trigonometric functions and fundamental constants. In this section we evaluate Eq (13) which involves symbolic manipulation to prove identities, find functional equations, or determine asymptotic behaviour. Interested readers can use this equation to derive new identities involing the Hurwitz-Lerch zeta function or to establish relationships between different special functions. The behaviour of the series as parameters like *n*, *m*, or *z* become large could be a focus of theoretical analysis. Such studies are common in analytic number theory and can reveal properties of the functions involved. The Hurwitz-Lerch function is defined by an infinite series, but it can be extended to a larger domain through analytical continuation. Eq (13) can be viewed as part of an analytical continuation or a formula that holds for specific ranges of parameters.

**Theorem 2.1.** *For all* $k,a\in \mathbb{C},-1<Re(m)<1,n\in Z_{+},z\in Z_{+}$ *then,*


$$\sum_{p=0}^{n}\left((-1{)}^{z+1}+1\right){\left((2z+1{)}^{p}\right)}^{k}e^{im(2z+1{)}^{p}}\Phi \left(-e^{2im(2z+1{)}^{p}},-k,\frac{\left(a(2z+1{)}^{-p}+1\right)}{2}\right)$$

$$+\sum_{p=0}^{n}\sum_{j=1}^{z}(-1{)}^{j+z+1}{\left((2z+1{)}^{p}\right)}^{k}e^{im(-2j+2z+1)(2z+1{)}^{p-1}}$$

$$(\Phi \left(-e^{2im(2z+1{)}^{p}},-k,\frac{1}{2}\left(a(2z+1{)}^{-p}+1-\frac{2j}{2z+1}\right)\right)$$

$$+e^{4ijm(2z+1{)}^{p-1}}\Phi \left(-e^{2im(2z+1{)}^{p}},-k,\frac{j}{2z+1}+\frac{1}{2}\left(a(2z+1{)}^{-p}+1\right)\right))$$

$$={\left((2z+1{)}^{n}\right)}^{k}e^{im(2z+1{)}^{n}}\Phi \left(-e^{2im(2z+1{)}^{n}},-k,\frac{1}{2}\left(a(2z+1{)}^{-n}+1\right)\right)$$

$$-{\left(\frac{1}{2z+1}\right)}^{k}e^{\frac{im}{2z+1}}\Phi \left(-e^{\frac{2im}{2z+1}},-k,\frac{1}{2}(2za+a+1)\right)$$ (13)

*Proof*: Observe that the addition of the right-hand sides of Eqs (9) and (10), is equal to the addition of the right-hand sides of Eqs (11) and (12) so we may equate the left-hand sides and simplify relative to Eq (1) and the Gamma function to yield the stated result. □

**Example 2.2.** *The degenerate case.*


$$\sum_{p=0}^{n}\sec\left(m(2z+1{)}^{p}\right)\left(\sum_{j=1}^{z}2e^{i\pi (j+z)}\cos\left(2jm(2z+1{)}^{p-1}\right)+e^{i\pi z}-1\right)$$

$$=\sec\left(\frac{m}{2z+1}\right)-\sec\left(m(2z+1{)}^{n}\right)$$ (14)

*Proof*: Use Eq (13) and set *k* = 0 and simplify using entry (2) in Table below Eq (64:12:7) in [34]. □

**Example 2.3.** *Double finite product of quotient gamma functions, where* $a\in \mathbb{C},n\in Z_{+}$*, z is even. Multiple finite products involving the gamma function are recorded in [p. 214] in [28] and [p.173] in [27].*


$$\prod_{p=0}^{n}\prod_{j=1}^{z}{\left(\frac{\Gamma \left(\frac{1}{4}\left(a(2z+1{)}^{-p}+1-\frac{2j}{2z+1}\right)\right)\Gamma \left(\frac{1}{4}\left(a(2z+1{)}^{-p}+1+\frac{2j}{2z+1}\right)\right)}{\Gamma \left(\frac{1}{4}\left(a(2z+1{)}^{-p}+3-\frac{2j}{2z+1}\right)\right)\Gamma \left(\frac{1}{4}\left(a(2z+1{)}^{-p}+3+\frac{2j}{2z+1}\right)\right)}\right)}^{(-1{)}^{j}}$$

$$=\frac{(2z+1{)}^{\frac{n+1}{2}}\Gamma \left(\frac{1}{4}(2za+a+1)\right)\Gamma \left(\frac{1}{4}\left(a(2z+1{)}^{-n}+3\right)\right)}{\Gamma \left(\frac{1}{4}(2za+a+3)\right)\Gamma \left(\frac{1}{4}\left(a(2z+1{)}^{-n}+1\right)\right)}$$ (15)

*Proof*: Use Eq (13) and set *m* = 0 and simplify in terms of the Hurwitz zeta function using entry (4) in Table below (64:12:7) in [34]. Next take the first partial derivative with respect to *k* and set *k* = 0 and simplify in terms of the log-gamma function using Eq (25.11.18) in [35]. Next we take the exponential of both sides and simplify in terms of the gamma function. □

**Example 2.4.** *Product of gamma functions in terms of the square root of odd numbers greater than equal to 5. We also present a product formula for the ratio of the Elliptic integral of the first kind see [p.1140] in [36].*


$$\frac{\Gamma \left(\frac{a+1}{4}\right)\Gamma \left(\frac{1}{4}(2za+a+3)\right)}{\Gamma \left(\frac{a+3}{4}\right)\Gamma \left(\frac{1}{4}(2za+a+1)\right)}\prod_{j=1}^{z}{\left(\frac{\Gamma \left(\frac{1}{4}\left(a-\frac{2j}{2z+1}+1\right)\right)\Gamma \left(\frac{1}{4}\left(a+\frac{2j}{2z+1}+1\right)\right)}{\Gamma \left(\frac{1}{4}\left(a-\frac{2j}{2z+1}+3\right)\right)\Gamma \left(\frac{1}{4}\left(a+\frac{2j}{2z+1}+3\right)\right)}\right)}^{(-1{)}^{j}}$$

$$\;=\sqrt{2z+1}$$ (16)

*Proof*: Use Eq (15) and set *p* = *n* = 0 and simplify. □

**Example 2.5.** *A Hurwitz-Lerch zeta functional equation with first parameter involving the cubic root of z.* Eq (17) *relates a function with a complex, fractional argument,* −(*iz*)^2/3^*, to a linear combination of several Hurwitz-Lerch zeta functions with arguments involving powers of z. The right-hand side is a combination of terms with arguments like z*^2^ *and z*^6^*, implying a complex relationship between the parameters and the overall functional behaviour.*


$$\Phi \left(-(iz{)}^{2/3},s,a\right)$$

$$=9^{-s}(3^{s}\Phi \left(z^{2},s,\frac{a}{3}\right)+(iz{)}^{2/3}(\Phi \left(z^{6},s,\frac{a+1}{9}\right)-2\times 3^{s}\Phi \left(z^{2},s,\frac{a+1}{3}\right)$$

$$+3^{s}(iz{)}^{2/3}\Phi \left(z^{2},s,\frac{a+2}{3}\right)+z^{4}\Phi \left(z^{6},s,\frac{a+7}{9}\right)+z^{2}\Phi \left(z^{6},s,\frac{a+4}{9}\right)))$$ (17)

*Proof*: *Use* Eq (13) *and set* $n=1,z=1,m=-i\log(z),a=e^{ia}$ *and simplify. Next replace* $a=\frac{2}{3}\left(a-\frac{1}{2}\right),k=-s,z=zi$ *and simplify.* □

**Example 2.6.** *Double finite sum in terms of trigonometric functions.* Eq (18) *slightly resembles a Fourier series due to the trigonometric functions and sums, but it is not a standard Fourier series. This equation’s structure involves a finite range of summation, a complex exponential with a phase of π, and terms where the argument of the trigonometric function depends on both summation indices p and j.*


$$\sum_{p=0}^{n}\sum_{j=1}^{z}2e^{i\pi (j+z)}(2z+1{)}^{p-1}\sec\left(x(2z+1{)}^{p}\right)$$

$$\left((2z+1)\tan\left(x(2z+1{)}^{p}\right)\cos\left(2jx(2z+1{)}^{p-1}\right)-2j\sin\left(2jx(2z+1{)}^{p-1}\right)\right)$$

$$+\sum_{p=0}^{n}\left(1-e^{i\pi z}\right)(2z+1{)}^{p}\tan\left(x(2z+1{)}^{p}\right)\sec\left(x(2z+1{)}^{p}\right)$$

$$=\frac{1}{8z+4}(\sec^{2}\left(\frac{x}{2z+1}\right)((2z+1{)}^{n+1}$$

$$(2\sin\left(x(2z+1{)}^{n}\right)-\sin\left(x\left(\frac{2}{2z+1}-(2z+1{)}^{n}\right)\right)$$

$$+\sin\left(x\left((2z+1{)}^{n}+\frac{2}{2z+1}\right)\right))-\sin\left(x\left(\frac{1}{2z+1}-2(2z+1{)}^{n}\right)\right)$$

$$-\sin\left(x\left(2(2z+1{)}^{n}+\frac{1}{2z+1}\right)\right)-2\sin\left(\frac{x}{2z+1}\right))\sec^{2}\left(x(2z+1{)}^{n}\right))$$ (18)

*Proof*: Use Eq (13) and set k=1,a=1,m=x and apply the method in section (8) in [26]. □

**Example 2.7.** *A functional equation involving the Hurwitz-Lerch zeta function. This equation connects a Hurwitz-Lerch function with the argument* $\left(-\sqrt[{3}]{-z}\right)$ *to a linear combination of three other Hurwitz-Lerch functions, where the argument is simply z. This relationship demonstrates how the function behaves under a scaling of its argument by a factor related to the cubic root. Similar functional equations were studied in the work by Apostol see Eq (2.6) in [37]. Apostol presented Riemann’s method and differential-difference equations in his work when studying functional equations for the Hurwitz-Lerch zeta function.*


$$\Phi \left(-\sqrt[{3}]{-z},s,a\right)$$

$$=3^{-s}\left(\Phi \left(z,s,\frac{a}{3}\right)-\sqrt[{3}]{-z}\Phi \left(z,s,\frac{a+1}{3}\right)+(-z{)}^{2/3}\Phi \left(z,s,\frac{a+2}{3}\right)\right)$$ (19)

*Proof*: Use Eq (13) and set $n=0,z=1,m=\log(z)/(2i),a=e^{ia}$ and simplify. Next replace $a=\frac{2}{3}\left(a-\frac{1}{2}\right),k=-s$ and simplify. □

**Example 2.8.** *Finite product involving the product of trigonometric functions. A multiplicative Identity Involving the Hurwitz-Lerch zeta function and parity filtering.*


$$\prod_{p=0}^{n}\prod_{j=1}^{z}\exp(i(-1{)}^{j+z}(2z+1{)}^{-p}e^{im(-2j+2z+1)(2z+1{)}^{p-1}}$$

$$\left(\Phi \left(-e^{2im(2z+1{)}^{p}},1,\frac{1}{2}-\frac{j}{2z+1}\right)+e^{4ijm(2z+1{)}^{p-1}}\Phi \left(-e^{2im(2z+1{)}^{p}},1,\frac{j}{2z+1}+\frac{1}{2}\right)\right))$$

$$\prod_{p=0}^{n}{\left(1-ie^{im(2z+1{)}^{p}}\right)}^{\left((-1{)}^{z+1}+1\right)(2z+1{)}^{-p}}{\left(1+ie^{im(2z+1{)}^{p}}\right)}^{\left((-1{)}^{z}-1\right)(2z+1{)}^{-p}}$$

$$={\left(i\cot\left(\frac{2m+2\pi z+\pi }{8z+4}\right)\right)}^{2z+1}{\left(-i\tan\left(\frac{1}{4}\left(2m(2z+1{)}^{n}+\pi \right)\right)\right)}^{(2z+1{)}^{-n}}$$ (20)

*Proof*: Use Eq (13) and set k=−1,a=1 and simplify using entry (3) in Table below (64:12:7) in [34]. □

### 2.1 Special cases involving the double finite sum of the Hurwitz-Lerch zeta function

In this section we derive special cases of the Hurwitz-Lerch zeta function in terms of special functions and Catalan’s constant *C*. The Hurwitz-Lerch zeta function is connected to the gamma function and polylogarithm, and special cases often involve these functions. For example, some derivations result in special cases involving infinite products of exponential functions that can be related to the gamma function or quotients of gamma functions. This happens because the integral representations and functional equations of these functions are connected.

**Example 2.9.** *Double finite sum in terms of Catalan’s constant C.*


$$\sum_{p=0}^{n}\sum_{j=1}^{z}(2z+1{)}^{-2p}(16C\left((-1{)}^{z}-1\right)+(-1{)}^{j+z}(\psi^{\left(1\right)}\left(\frac{-2j+2z+1}{8z+4}\right)$$

$$+\psi^{\left(1\right)}\left(\frac{2j+2z+1}{8z+4}\right)-\psi^{\left(1\right)}\left(\frac{-2j+6z+3}{8z+4}\right)-\psi^{\left(1\right)}\left(\frac{2j+6z+3}{8z+4}\right)))$$

$$=16C\left((2z+1{)}^{2}-(2z+1{)}^{-2n}\right)$$ (21)

*Proof*: Use Eq (13) and set k=−2,a=1,m=0 and simplify using Eq (4) in [38]. □

**Example 2.10.** *Finite sum in terms of Catalan’s constant C and the digamma function* $\psi^{\left(1\right)}(s)$.


$$\sum_{j=1}^{z}\frac{(2z+1{)}^{2}(-1{)}^{j+z}}{4z(z+1)}(\psi^{\left(1\right)}\left(\frac{-2j+2z+1}{8z+4}\right)+\psi^{\left(1\right)}\left(\frac{2j+2z+1}{8z+4}\right)$$

$$-\psi^{\left(1\right)}\left(\frac{-2j+6z+3}{8z+4}\right)-\psi^{\left(1\right)}\left(\frac{2j+6z+3}{8z+4}\right))$$

$$=\frac{4C(2z+1{)}^{2}\left(4z-(-1{)}^{z}+5\right)}{z+1}$$ (22)

*Proof*: Use Eq (21) and take the limit as n→∞ and simplify. □

**Example 2.11.** *Finite sum in terms of the first partial derivative of the Hurwitz-Lerch zeta function.*


$$\sum_{j=1}^{z}(-1{)}^{j}\left(\Phi^{\prime }\left(-1,-1,\frac{1}{2}-\frac{j}{2z+1}\right)+\Phi^{\prime }\left(-1,-1,\frac{j}{2z+1}+\frac{1}{2}\right)\right)$$

$$=\frac{C\left(-2z+(-1{)}^{-z}-1\right)}{\pi (2z+1)}$$ (23)

*Proof*: Use Eq (13) and take the first partial derivative with respect to *k* and set k=1,m=0,a=1 and simplify using Eq (20) in [38]. □

**Example 2.12.** *Double finite sum involving Euler’s constant γ. Euler’s constant arises in the expansion of the gamma function, special values of the digamma function, and expressions for the Riemann zeta function.*


$$\sum_{p=0}^{n}\sum_{j=1}^{z}2ie^{i\pi (j+z)}(2z+1{)}^{-p}(-\Phi^{\prime }\left(-1,1,\frac{1}{2}-\frac{j}{2z+1}\right)-\Phi^{\prime }\left(-1,1,\frac{j}{2z+1}+\frac{1}{2}\right)$$

$$+\pi \sec\left(\frac{\pi j}{2z+1}\right)\log\left(i(2z+1{)}^{p}\right))-i\pi \left((-1{)}^{z}-1\right)\sum_{p=0}^{n}(2z+1{)}^{-p}\log\left(-\frac{ie^{\gamma }\pi^{3}(2z+1{)}^{-p}}{32\Gamma {\left(\frac{5}{4}\right)}^{4}}\right)$$

$$=-i\pi (2z+1{)}^{-n}((2z+1{)}^{n+1}\log\left(-\frac{8ie^{\gamma }\pi^{3}(2z+1)}{\Gamma {\left(\frac{1}{4}\right)}^{4}}\right)$$

$$+\log\left(\frac{32ie^{-\gamma }\Gamma {\left(\frac{5}{4}\right)}^{4}(2z+1{)}^{n}}{\pi^{3}}\right))$$ (24)

*Proof*: Use Eq (13) and take the first partial derivative with respect to *k* and set k=−1,m=0,a=1 and simplify using Eq (53) in [38]. □

**Example 2.13.** *The exponential of the Hurwitz-Lerch zeta function in terms of the log-gamma function. This identity is an example of how seemingly different mathematical constants, the derivative of a generalized zeta function, Euler’s constant, the Gamma function, and π are interconnected. It is a good example of the structure inherent in the theory of special functions and demonstrates the usefulness of complex analysis in deriving and proving such unexpected relationships.*

$$\exp\left(-\frac{13\left(\Phi^{\prime }\left(-1,1,\frac{1}{6}\right)+\Phi^{\prime }\left(-1,1,\frac{5}{6}\right)\right)}{54\pi }\right)=\frac{2^{11/27}e^{-13\gamma /27}\Gamma \left(\frac{1}{4}\right)\Gamma {\left(\frac{5}{4}\right)}^{25/27}}{3^{13/36}\pi^{13/9}}$$ (25)

*Proof*: Use Eq (24) and set n=2,z=1 and simplify. □

**Example 2.14.** *A double finite sum involving Catalan’s constant C.*


$$\sum_{p=0}^{n}4\left((-1{)}^{z}-1\right)(2z+1{)}^{-2p}\left(-\Phi^{\prime }\left(-1,2,\frac{1}{2}\right)+4C\log\left(i(2z+1{)}^{p}\right)\right)$$

$$+\sum_{p=0}^{n}\sum_{j=1}^{z}(-1{)}^{j+z}(2z+1{)}^{-2p}(-4\Phi^{\prime }\left(-1,2,\frac{1}{2}-\frac{j}{2z+1}\right)-4\Phi^{\prime }\left(-1,2,\frac{j}{2z+1}+\frac{1}{2}\right)$$

$$+(\psi^{\left(1\right)}\left(\frac{-2j+2z+1}{8z+4}\right)+\psi^{\left(1\right)}\left(\frac{2j+2z+1}{8z+4}\right)-\psi^{\left(1\right)}\left(\frac{-2j+6z+3}{8z+4}\right)$$

$$-\psi^{\left(1\right)}\left(\frac{2j+6z+3}{8z+4}\right))\log\left(i(2z+1{)}^{p}\right))$$

$$=4(2z+1{)}^{-2n}\left(\Phi^{\prime }\left(-1,2,\frac{1}{2}\right)-4C\log\left(i(2z+1{)}^{n}\right)\right)$$

$$+4(2z+1{)}^{2}\left(-\Phi^{\prime }\left(-1,2,\frac{1}{2}\right)+4C\log\left(\frac{i}{2z+1}\right)\right)$$ (26)

*Proof*: Use Eq (13) and take the first partial derivative with respect to *k* and set k=−2,m=0,a=1 and simplify using Eq (4) in [38]. □

**Example 2.15.** *Double finite product involving tangent and cotangent functions.*


$$\prod_{p=0}^{n}\prod_{j=1}^{z}\exp\left(\frac{\pi i(-1{)}^{j+z}\sec\left(\frac{j\pi }{1+2z}\right)}{(1+2z{)}^{p}}\right)\prod_{p=0}^{n}\tan^{\frac{-1+(-1{)}^{z}}{(1+2z{)}^{p}}}\left(\frac{1}{4}\pi \left(1+2(1+2z{)}^{1+p}\right)\right)$$

$$=i{\left(-(-1{)}^{3/4}\right)}^{\frac{(1+2z{)}^{-n}\left(-1+(-1{)}^{z}-\left(-1+(-1{)}^{z}\right)(1+2z{)}^{1+n}\right)}{z}}$$

$$(-1{)}^{z}{\left(i\cot\left(\frac{1}{4}\pi \left(1+2(1+2z{)}^{1+n}\right)\right)\right)}^{(1+2z{)}^{-n}}$$ (27)

*Proof*: Use Eq (13) and set k=−1,m=π(2z+1),a=1 and simplify using entry (1) in Table below (64:12:7) in [34]. □

**Example 2.16.** *Double finite product involving the tangent function.*


$$\prod_{p=0}^{n}\prod_{j=1}^{z}\exp(-\frac{i(-1{)}^{j+z}}{(1+2z{)}^{p}}(e^{i\pi (1+2z{)}^{-1-n+p}(1-2j+2z)}\Phi \left(-e^{2i\pi (1+2z{)}^{-n+p}},1,\frac{1}{2}-\frac{j}{1+2z}\right)$$

$$+e^{i\pi (1+2z{)}^{-1-n+p}(1+2j+2z)}\Phi \left(-e^{2i\pi (1+2z{)}^{-n+p}},1,\frac{1}{2}+\frac{j}{1+2z}\right)))$$

$$\prod_{p=0}^{n}{\left(-i\tan\left(\frac{1}{4}\pi \left(1+2(1+2z{)}^{-n+p}\right)\right)\right)}^{\frac{-1+(-1{)}^{z}}{(1+2z{)}^{p}}}$$

$$=-e^{-\frac{\pi i}{2(1+2z{)}^{n}}}{\left(-i\tan\left(\frac{1}{4}\pi \left(1+2(1+2z{)}^{-1-n}\right)\right)\right)}^{2z}\left(i\tan\left(\frac{1}{4}\pi \left(1+2(1+2z{)}^{-1-n}\right)\right)\right)$$ (28)

*Proof*: Use Eq (13) and set $k=-1,m=\pi (2z\;+\;1{)}^{-n},a=1$ and simplify using entry (1) in Table below (64:12:7) in [34]. □

**Example 2.17.** *A double finite product involving the gamma function and table of special cases.*


$$\prod_{p=0}^{n}\prod_{j=1}^{z}{\left(i(2z+1{)}^{p}\right)}^{(-1{)}^{j+z}+\frac{1}{2}\left((-1{)}^{z}-1\right)}{\left(\frac{\Gamma \left(\frac{-2j+6z+3}{8z+4}\right)\Gamma \left(\frac{j}{4z+2}+\frac{3}{4}\right)}{\Gamma \left(\frac{-2j+2z+1}{8z+4}\right)\Gamma \left(\frac{j}{4z+2}+\frac{1}{4}\right)}\right)}^{(-1{)}^{j+z}}$$

$$=(2z+1{)}^{\frac{1}{2}(-n-1)}{\left(\frac{3\Gamma \left(-\frac{3}{4}\right)}{\Gamma \left(-\frac{1}{4}\right)}\right)}^{(-1{)}^{z}-1}$$ (29)

*Proof*: Use Eq (13) and set m=0,a=1 and simplify in terms of the Hurwitz zeta function using entry (4) in Table below (64:12:7) in [34]. Next take the first partial derivative with respect to *k* and set *k* = 0 and simplify in terms of the log-gamma function using Eq (25.11.18) in [35]. □

#### 2.1.1 Table of quotient gamma functions.

In this section we look at simplified forms of Eq (29). This table of quotient gamma functions is not a static catalog of numerical values but rather a collection of identities and formulas that express the ratio of two gamma functions, $\frac{\Gamma (z+a)}{\Gamma (z+b)}$, in terms of other functions, special values, or asymptotic expansions. These relations are important in mathematics and prove useful in analytic number theory and probability theory.

**Example 2.18.** *A Gosper relation for a q-trigonometric form in terms of quotient gamma functions given on page (80) in [39] and entry (1) in* Table 1.


$$\prod_{n=0}^{\infty }\frac{\left(10n+1)(10n+3)(10n+7)(10n+9\right)}{\left(10n+2)(10n+4)(10n+6)(10n+8\right)}=\frac{\sin\left(\frac{3\pi }{10}\right)}{2\sin^{2}\left(\frac{2\pi }{5}\right)}$$

$$=\frac{\Gamma \left(\frac{3}{20}\right)\Gamma \left(\frac{7}{20}\right)\Gamma \left(\frac{11}{20}\right)\Gamma \left(\frac{19}{20}\right)}{\Gamma \left(\frac{1}{20}\right)\Gamma \left(\frac{9}{20}\right)\Gamma \left(\frac{13}{20}\right)\Gamma \left(\frac{17}{20}\right)}=\frac{1}{\sqrt{5}}$$ (30)

**Table 1:** Table of quotient gamma functions.

$\frac{\Gamma \left(\frac{3}{20}\right)\Gamma \left(\frac{7}{20}\right)\Gamma \left(\frac{11}{20}\right)\Gamma \left(\frac{19}{20}\right)}{\Gamma \left(\frac{1}{20}\right)\Gamma \left(\frac{9}{20}\right)\Gamma \left(\frac{13}{20}\right)\Gamma \left(\frac{17}{20}\right)}$	$\frac{1}{\sqrt{5}}$
$\frac{\Gamma (\frac{3}{20}{)}^{2}\Gamma (\frac{7}{20}{)}^{2}\Gamma (\frac{11}{20}{)}^{2}\Gamma (\frac{19}{20}{)}^{2}}{\Gamma (\frac{1}{20}{)}^{2}\Gamma (\frac{9}{20}{)}^{2}\Gamma (\frac{13}{20}{)}^{2}\Gamma (\frac{17}{20}{)}^{2}}$	$\frac{1}{5}$
$\frac{\Gamma (\frac{1}{12}{)}^{2}\Gamma (\frac{7}{36}{)}^{2}\Gamma (\frac{11}{36}{)}^{2}\Gamma (\frac{5}{12}{)}^{2}\Gamma (\frac{19}{36}{)}^{2}\Gamma (\frac{23}{36}{)}^{2}\Gamma (\frac{31}{36}{)}^{2}\Gamma (\frac{35}{36}{)}^{2}}{\Gamma (\frac{1}{36}{)}^{2}\Gamma (\frac{5}{36}{)}^{2}\Gamma (\frac{13}{36}{)}^{2}\Gamma (\frac{17}{36}{)}^{2}\Gamma (\frac{7}{12}{)}^{2}\Gamma (\frac{25}{36}{)}^{2}\Gamma (\frac{29}{36}{)}^{2}\Gamma (\frac{11}{12}{)}^{2}}$	$\frac{1}{9}$
$\frac{\Gamma (\frac{1}{12}{)}^{3}\Gamma (\frac{7}{36}{)}^{3}\Gamma (\frac{11}{36}{)}^{3}\Gamma (\frac{5}{12}{)}^{3}\Gamma (\frac{19}{36}{)}^{3}\Gamma (\frac{23}{36}{)}^{3}\Gamma (\frac{31}{36}{)}^{3}\Gamma (\frac{35}{36}{)}^{3}}{\Gamma (\frac{1}{36}{)}^{3}\Gamma (\frac{5}{36}{)}^{3}\Gamma (\frac{13}{36}{)}^{3}\Gamma (\frac{17}{36}{)}^{3}\Gamma (\frac{7}{12}{)}^{3}\Gamma (\frac{25}{36}{)}^{3}\Gamma (\frac{29}{36}{)}^{3}\Gamma (\frac{11}{12}{)}^{3}}$	$\frac{1}{27}$
$\frac{\Gamma (\frac{1}{12}{)}^{4}\Gamma (\frac{7}{36}{)}^{4}\Gamma (\frac{11}{36}{)}^{4}\Gamma (\frac{5}{12}{)}^{4}\Gamma (\frac{19}{36}{)}^{4}\Gamma (\frac{23}{36}{)}^{4}\Gamma (\frac{31}{36}{)}^{4}\Gamma (\frac{35}{36}{)}^{4}}{\Gamma (\frac{1}{36}{)}^{4}\Gamma (\frac{5}{36}{)}^{4}\Gamma (\frac{13}{36}{)}^{4}\Gamma (\frac{17}{36}{)}^{4}\Gamma (\frac{7}{12}{)}^{4}\Gamma (\frac{25}{36}{)}^{4}\Gamma (\frac{29}{36}{)}^{4}\Gamma (\frac{11}{12}{)}^{4}}$	$\frac{1}{81}$
$-\frac{\Gamma \left(\frac{3}{28}\right)\Gamma \left(\frac{11}{28}\right)\Gamma \left(\frac{15}{28}\right)\Gamma \left(\frac{19}{28}\right)\Gamma \left(\frac{23}{28}\right)\Gamma \left(\frac{27}{28}\right)}{\Gamma \left(\frac{1}{28}\right)\Gamma \left(\frac{5}{28}\right)\Gamma \left(\frac{9}{28}\right)\Gamma \left(\frac{13}{28}\right)\Gamma \left(\frac{17}{28}\right)\Gamma \left(\frac{25}{28}\right)}$	$\frac{\Gamma (-\frac{1}{4}{)}^{2}}{9\sqrt{7}\Gamma (-\frac{3}{4}{)}^{2}}$
$\frac{\Gamma (\frac{3}{20}{)}^{3}\Gamma (\frac{7}{20}{)}^{3}\Gamma (\frac{11}{20}{)}^{3}\Gamma (\frac{19}{20}{)}^{3}}{\Gamma (\frac{1}{20}{)}^{3}\Gamma (\frac{9}{20}{)}^{3}\Gamma (\frac{13}{20}{)}^{3}\Gamma (\frac{17}{20}{)}^{3}}$	$\frac{1}{5\sqrt{5}}$
$\frac{\Gamma (\frac{3}{20}{)}^{4}\Gamma (\frac{7}{20}{)}^{4}\Gamma (\frac{11}{20}{)}^{4}\Gamma (\frac{19}{20}{)}^{4}}{\Gamma (\frac{1}{20}{)}^{4}\Gamma (\frac{9}{20}{)}^{4}\Gamma (\frac{13}{20}{)}^{4}\Gamma (\frac{17}{20}{)}^{4}}$	$\frac{1}{25}$
$\frac{\Gamma (\frac{3}{20}{)}^{5}\Gamma (\frac{7}{20}{)}^{5}\Gamma (\frac{11}{20}{)}^{5}\Gamma (\frac{19}{20}{)}^{5}}{\Gamma (\frac{1}{20}{)}^{5}\Gamma (\frac{9}{20}{)}^{5}\Gamma (\frac{13}{20}{)}^{5}\Gamma (\frac{17}{20}{)}^{5}}$	$\frac{1}{25\sqrt{5}}$
$\frac{\Gamma (\frac{1}{12}{)}^{6}\Gamma (\frac{7}{36}{)}^{6}\Gamma (\frac{11}{36}{)}^{6}\Gamma (\frac{5}{12}{)}^{6}\Gamma (\frac{19}{36}{)}^{6}\Gamma (\frac{23}{36}{)}^{6}\Gamma (\frac{31}{36}{)}^{6}\Gamma (\frac{35}{36}{)}^{6}}{\Gamma (\frac{1}{36}{)}^{6}\Gamma (\frac{5}{36}{)}^{6}\Gamma (\frac{13}{36}{)}^{6}\Gamma (\frac{17}{36}{)}^{6}\Gamma (\frac{7}{12}{)}^{6}\Gamma (\frac{25}{36}{)}^{6}\Gamma (\frac{29}{36}{)}^{6}\Gamma (\frac{11}{12}{)}^{6}}$	$\frac{1}{729}$

**Example 2.19.** *Double finite product involving exponential of the Hurwitz-Lerch zeta function and quotient tangent functions.*


$$\prod_{p=0}^{n}\prod_{j=1}^{z}\exp(\frac{i(-1{)}^{j+z}}{(1+2z{)}^{p}}(e^{im(1+2z{)}^{-1+p}(1-2j+2z)}\Phi \left(-e^{2im(1+2z{)}^{p}},1,\frac{1}{2}-\frac{j}{1+2z}\right)$$

$$+e^{im(1+2z{)}^{-1+p}(1+2j+2z)}\Phi \left(-e^{2im(1+2z{)}^{p}},1,\frac{1}{2}+\frac{j}{1+2z}\right)$$

$$-e^{ir(1+2z{)}^{-1+p}(1-2j+2z)}\Phi \left(-e^{2ir(1+2z{)}^{p}},1,\frac{1}{2}-\frac{j}{1+2z}\right)$$

$$-e^{ir(1+2z{)}^{-1+p}(1+2j+2z)}\Phi \left(-e^{2ir(1+2z{)}^{p}},1,\frac{1}{2}+\frac{j}{1+2z}\right)))$$

$$\prod_{p=0}^{n}{\left(\frac{\tan\left(\frac{1}{4}\left(\pi +2m(1+2z{)}^{p}\right)\right)}{\tan\left(\frac{1}{4}\left(\pi +2r(1+2z{)}^{p}\right)\right)}\right)}^{-\left(\left(-1+(-1{)}^{z}\right)(1+2z{)}^{-p}\right)}$$

$$={\left(\frac{\tan\left(\frac{2m+\pi +2\pi z}{4+8z}\right)}{\tan\left(\frac{\pi +2r+2\pi z}{4+8z}\right)}\right)}^{-1-2z}{\left(\frac{\tan\left(\frac{1}{4}\left(\pi +2m(1+2z{)}^{n}\right)\right)}{\tan\left(\frac{1}{4}\left(\pi +2r(1+2z{)}^{n}\right)\right)}\right)}^{(1+2z{)}^{-n}}$$ (31)

*Proof*: Use Eq (13) and form a second equation by replacing m→r and take the difference of both these equations then set k=−1,a=1 and simplify using entry (3) of Section (64:12) in [34]. □

**Example 2.20.** *Finite product involving exponential of the Hurwitz-Lerch zeta function and quotient tangent functions.*


$$\prod_{j=1}^{z}\exp(i(-1{)}^{j+z}(e^{im\left(1-\frac{2j}{1+2z}\right)}\Phi \left(-e^{2im},1,\frac{1}{2}-\frac{j}{1+2z}\right)$$

$$+e^{im\left(1+\frac{2j}{1+2z}\right)}\Phi \left(-e^{2im},1,\frac{1}{2}+\frac{j}{1+2z}\right)-e^{ir\left(1-\frac{2j}{1+2z}\right)}\Phi \left(-e^{2ir},1,\frac{1}{2}-\frac{j}{1+2z}\right)$$

$$-e^{ir\left(1+\frac{2j}{1+2z}\right)}\Phi \left(-e^{2ir},1,\frac{1}{2}+\frac{j}{1+2z}\right)))$$

$$=\frac{\tan\left(\frac{1}{4}(2m+\pi )\right){\left(\frac{\tan\left(\frac{2m+\pi +2\pi z}{4+8z}\right)}{\tan\left(\frac{\pi +2r+2\pi z}{4+8z}\right)}\right)}^{-1-2z}}{\tan\left(\frac{1}{4}(\pi +2r)\right){\left(\frac{\tan\left(\frac{1}{4}(2m+\pi )\right)}{\tan\left(\frac{1}{4}(\pi +2r)\right)}\right)}^{1-(-1{)}^{z}}}$$ (32)

*Proof*: Use Eq (31) and set *p* = *n* = 0 and simplify, where *z* is any positive integer. □

### 2.2 An example involving the exponential of the hypergeometric function in terms of reciprocal angles

In this section we evaluate a function involving the exponential of a hypergeometric function in terms of reciprocal angles. This equation type is found in the theory of conformal mapping and modular forms, specifically involving the mapping properties of the Gauss hypergeometric function.

**Example 2.21.** *Exponential of the hypergeometric function involving reciprocal angles.*


$$\exp(i(6e^{\frac{i}{3}/r}{\;}_{2}F_{1}\left(\frac{1}{6},1;\frac{7}{6};-e^{2i/r}\right)-6e^{\frac{ir}{3}}{\;}_{2}F_{1}\left(\frac{1}{6},1;\frac{7}{6};-e^{2ir}\right)$$

$$+\frac{6}{5}e^{\frac{5i}{3}/r}{\;}_{2}F_{1}\left(\frac{5}{6},1;\frac{11}{6};-e^{2i/r}\right)-\frac{6}{5}e^{\frac{5ir}{3}}{\;}_{2}F_{1}\left(\frac{5}{6},1;\frac{11}{6};-e^{2ir}\right)))$$

$$=\frac{\tan\left(\frac{1}{4}(2r+\pi )\right)\tan^{3}\left(\frac{1}{12}(2r+3\pi )\right)}{\tan\left(\frac{1}{4}\left(\frac{2}{r}+\pi \right)\right)\tan^{3}\left(\frac{1}{12}\left(\frac{2}{r}+3\pi \right)\right)}$$ (33)

*Proof*: Use Eq (32) and replace *m* by 1/*r* and set *z* = 1 and simplify. □

**Example 2.22.** *In this example we look at the contour plot of the right-hand side of* Eq (33)*. This plot is a 3D plot of* $\left|\frac{\tan^{3}\left(\frac{\pi }{4}+\frac{r}{6}\right)\tan\left(\frac{1}{4}(\pi +2r)\right)}{\tan^{3}\left(\frac{\pi }{4}+\frac{1}{6r}\right)\tan\left(\frac{1}{4}\left(\pi +\frac{2}{r}\right)\right)}\right|$ *over the complex rectangle with corners *z**_*min*_ *and *z**_*max*_*. In this plot we see a point where the function is zero, appearing as a low point or a “valley” in the plot. The colours will cycle counter-clockwise around a zero. There exists a pole shown by a high spike or “mountain” in the plot. The colours will cycle clockwise around a pole.*

### 2.3 Determining the first partial derivative of the Hurwitz-Lerch zeta function

In this example we derive a finite sum and product expressions which can be used to determine the first partial derivatives of the Hurwitz-Lerch zeta function. These derivatives are with respect to the first and the second parameters and are set equal zero. These expressions are particularly useful in simplifying definite integrals whose closed form solution is in terms of the Hurwitz-Lerch zeta function.

**Example 2.23** *Finite sum involving the Hurwitz zeta function.*


$$\sum_{j=1}^{z}(-1{)}^{j+z}e^{i\pi \left(1-\frac{2j}{2z+1}\right)}$$

$$(\zeta \left(-k,\frac{a-2j+2(a+3)z+3}{8z+4}\right)-\zeta \left(-k,\frac{1}{4}\left(a-\frac{2j}{2z+1}+1\right)\right)$$

$$+e^{\frac{4i\pi j}{2z+1}}\left(\zeta \left(-k,\frac{a+2j+2(a+3)z+3}{8z+4}\right)-\zeta \left(-k,\frac{1}{4}\left(a+\frac{2j}{2z+1}+1\right)\right)\right))$$

$$=-\left((-1{)}^{z}-1\right)\left(\zeta \left(-k,\frac{a+1}{4}\right)-\zeta \left(-k,\frac{a+3}{4}\right)\right)+2^{-k}(2^{k}(\zeta \left(-k,\frac{a+3}{4}\right)$$

$$-\zeta \left(-k,\frac{a+1}{4}\right))-e^{\frac{i\pi }{2z+1}}{\left(\frac{1}{2z+1}\right)}^{k}\Phi \left(-e^{\frac{2i\pi }{2z+1}},-k,\frac{1}{2}(2za+a+1)\right))$$ (34)

*Proof*: Use Eq (13) and set p=n=0,m=π and simplify in terms of the Hurwitz zeta function. □

**Example 2.24.** *A generalized formula for the first partial derivative of the Hurwitz-Lerch zeta function in terms of the log-gamma function.*


$$\sum_{j=1}^{z}(-1{)}^{j+z}e^{-\frac{2i\pi (j-z)}{2z+1}}(\log\left(\frac{\Gamma \left(\frac{1}{4}\left(a-\frac{2j}{2z+1}+1\right)\right)}{\Gamma \left(\frac{1}{4}\left(a-\frac{2j}{2z+1}+3\right)\right)}\right)$$

$$+e^{\frac{4i\pi j}{2z+1}}\log\left(\frac{\Gamma \left(\frac{1}{4}\left(a+\frac{2j}{2z+1}+1\right)\right)}{\Gamma \left(\frac{1}{4}\left(a+\frac{2j}{2z+1}+3\right)\right)}\right))$$

$$-e^{-\frac{i\pi }{2z+1}}\log\left((4z+2{)}^{\frac{1}{2}\sec\left(\frac{\pi }{2z+1}\right)}{\left(\frac{\Gamma \left(\frac{a+1}{4}\right)}{\Gamma \left(\frac{a+3}{4}\right)}\right)}^{(-1{)}^{z}}\right)$$

$$=\Phi^{\prime }\left(-e^{\frac{2i\pi }{2z+1}},0,\frac{1}{2}(2az+a+1)\right)$$ (35)

*Proof*: Use Eq (13) and set p=n=0,m=π and simplify in terms of the Hurwitz zeta function. Next take the first partial derivative with respect to *k* and set *k* = 0 and simplify in terms of the log-gamma function. □

**Example 2.25.** *Finite product of quotient gamma functions raised to complex power.*


$$\prod_{j=1}^{z}{\left(\frac{\Gamma \left(\frac{1}{4}\left(a-\frac{2j}{2z+1}+3\right)\right)}{\Gamma \left(\frac{1}{4}\left(a-\frac{2j}{2z+1}+1\right)\right)}\right)}^{(-1{)}^{j+z}e^{-\frac{2i\pi j}{2z+1}}}{\left(\frac{\Gamma \left(\frac{1}{4}\left(a+\frac{2j}{2z+1}+3\right)\right)}{\Gamma \left(\frac{1}{4}\left(a+\frac{2j}{2z+1}+1\right)\right)}\right)}^{(-1{)}^{j+z}e^{\frac{2i\pi j}{2z+1}}}$$

$$=(4z+2{)}^{\frac{1}{2}\sec\left(\frac{\pi }{2z+1}\right)}{\left(\frac{\Gamma \left(\frac{a+1}{4}\right)}{\Gamma \left(\frac{a+3}{4}\right)}\right)}^{(-1{)}^{z}}\exp\left(e^{\frac{i\pi }{2z+1}}\Phi^{\prime }\left(-e^{\frac{2i\pi }{2z+1}},0,\frac{1}{2}(2az+a+1)\right)\right)$$ (36)

*Proof*: Use Eq (35) and take the exponential of both sides and simplify. Here *z* is any positive integer greater than or equal to 1. □

**Example 2.26.** *An example in terms of the log-gamma function.*

$$-(-1{)}^{2/3}\log\left(\frac{\Gamma \left(\frac{3}{8}\right){\left(\frac{\Gamma \left(\frac{5}{24}\right)}{\Gamma \left(\frac{17}{24}\right)}\right)}^{\sqrt[{3}]{-1}}{\left(\frac{\Gamma \left(\frac{25}{24}\right)}{\Gamma \left(\frac{13}{24}\right)}\right)}^{(-1{)}^{2/3}}}{6\Gamma \left(\frac{7}{8}\right)}\right)=\Phi^{\prime }\left(-(-1{)}^{2/3},0,\frac{5}{4}\right)$$ (37)

*Proof*: Use Eq (36) and set a=1/2,z=1 and simplify. □

**Example 2.27.** *A Complex Mellin-Barnes Type Integral Involving Nested Logarithms and Parameters. In this example we look at how* Eq (37) *can be applied to simplifying an infinite integral involving a nested logarithm function in terms of the log-gamma function. Here we use Eq (7) in [40] and take the first partial derivative with respect to k and set* $k=0,a=e^{\frac{3i\pi }{2n}}\alpha^{\frac{1}{n}},m=m+n/2$ *and simplify using* Eq (37)*. Finally we replace* n→v,y→x,α→b.


$$\int_{0}^{\infty }\frac{x^{-1+\frac{5v}{6}}\log\left(\log\left(b^{1/v}e^{\frac{3i\pi }{2v}}x\right)\right)}{1+bx^{v}}\;dx$$

$$=\frac{2\pi \log\left(\frac{2i\pi }{v}\right)+\left(-1-i\sqrt{3}\right)\pi \Phi^{\prime }\left(-(-1{)}^{2/3},0,\frac{5}{4}\right)}{b^{5/6}v}$$

$$=\frac{2\pi \log\left(\frac{2i\pi }{v}\right)+\left(-1-i\sqrt{3}\right)\pi \left(-(-1{)}^{2/3}\log\left(\frac{\Gamma \left(\frac{3}{8}\right){\left(\frac{\Gamma \left(\frac{5}{24}\right)}{\Gamma \left(\frac{17}{24}\right)}\right)}^{\sqrt[{3}]{-1}}{\left(\frac{\Gamma \left(\frac{25}{24}\right)}{\Gamma \left(\frac{13}{24}\right)}\right)}^{(-1{)}^{2/3}}}{6\Gamma \left(\frac{7}{8}\right)}\right)\right)}{b^{5/6}v}$$ (38)

*where Re*(*v*) > *1 and there exists a singularity at* $x=e^{-\frac{3i\pi }{2v}}b^{-1/v}$ *when Re*(*b*) > *0.*

**Example 2.28.** *Evaluation of an improper integral involving a nested logarithm and a rational function. In this example we use* Eq (38) *with* b=−1,v=4 *simplify and plot the singularities over the real and imaginary parts of the integrand.*


$$\int_{0}^{\infty }\frac{x^{7/3}\log\left(\log\left((-1{)}^{5/8}x\right)\right)}{1-x^{4}}\;dx$$

$$=\frac{1}{4}\sqrt[{6}]{-1}\pi \left(-2\log\left(\frac{i\pi }{2}\right)+\sqrt[{6}]{-1}\left(-i+\sqrt{3}\right)\log\left(\frac{\Gamma \left(\frac{3}{8}\right){\left(\frac{\Gamma \left(\frac{5}{24}\right)}{\Gamma \left(\frac{17}{24}\right)}\right)}^{\sqrt[{3}]{-1}}{\left(\frac{\Gamma \left(\frac{25}{24}\right)}{\Gamma \left(\frac{13}{24}\right)}\right)}^{(-1{)}^{2/3}}}{6\Gamma \left(\frac{7}{8}\right)}\right)\right)$$ (39)

*where there exists a singularity at x* = −*i*/2 *and we assume the value of the integral is a Cauchy principal value see [DLMF,* 19.17.8*]*

**Example 2.29.** *In this example we plot the real part of the right-hand side of* Eq (39)*. There exists a vertical asymptote at x* = 1*. Since there is a* (−1)^5/8^ *term in the argument of the logarithm then the nested logarithm term is always complex.*

**Example 2.30.** *In this example we plot the imaginary part of the right-hand side of* Eq (39)*. There exists a vertical asymptote at x* = 1*. Since there is a* (−1)^5/8^ *term in the argument of the logarithm then the nested logarithm term is always complex.*

### 2.4 Special cases involving fundamental constants

In this section we will evaluate Eq (34) and derive formulae in terms of fundamental constants; namely Catalan’s constant *C* given in Eqs (20) and (21) in [38], Glaisher’s constant *A*, given in Eq (18) in [38], Apery’s constant ζ(3), given in Eq (19). In combinatorics, *G* appears in enumerative problems, such as counting domino tilings on grid graphs, where it provides insight into the asymptotic behaviour of the number of possible tilings. In number theory, it is seen in a still-unproven conjecture related to primes of the form *n*^2^ + 1, implying a link between this irrational number and the distribution of prime numbers. In topology, Catalan’s constant has been shown to be related to the hyperbolic volume of the complement of certain links, providing a concrete mathematical invariant for understanding the geometry of three-dimensional spaces. Finally, in astrophysics, *G* appears in calculations involving the mass distribution of spiral galaxies, involving gravitational models. The Glaisher–Kinkelin constant is related to special functions like the Barnes *G*-function and the gamma function. The Glaisher constant emerges from an approximation involving the hyperfactorial, which is the product of integers raised to their own powers. The constant appears in a variety of integrals and sums, particularly those involving the gamma and Riemann zeta functions. It is also connected to the solution of Painlevé differential equations and plays a role in regularization techniques in quantum field theory and statistical mechanics, where it can manage divergences in calculations. Apery’s constant ζ(3) appears in various field in mathematics and physics, including the gamma function, special values of polylogarithms, and the Hurwitz-Lerch function. In physics, Apéry’s constant is involved in calculations related to quantum electrodynamics, particularly in determining the electron’s gyromagnetic ratio, as well as in the study of three-dimensional magnetic fields.

**Example 2.31.** *Finite sum in terms of Catalan’s constant.*


$$\sum_{j=1}^{z}(-1{)}^{j+z}e^{-\frac{2i\pi (j-z)}{2z+1}}(\psi^{\left(1\right)}\left(\frac{-2j+2z+1}{8z+4}\right)-\psi^{\left(1\right)}\left(\frac{-2j+6z+3}{8z+4}\right)$$

$$+e^{\frac{4i\pi j}{2z+1}}\left(\psi^{\left(1\right)}\left(\frac{2j+2z+1}{8z+4}\right)-\psi^{\left(1\right)}\left(\frac{2j+6z+3}{8z+4}\right)\right))$$

$$=16C(-1{)}^{z}e^{-\frac{i\pi }{2z+1}}+4(2z+1{)}^{2}\Phi \left(-e^{\frac{2i\pi }{2z+1}},2,\frac{1}{2}\right)$$ (40)

*Proof*: Use Eq (34) and set k=−2,a=0 and simplify. □

**Example 2.32.** *Finite sum in terms of Glaisher’s constant.*


$$\sum_{j=1}^{z}(-1{)}^{j+z}e^{i\pi \left(1-\frac{2j}{2z+1}\right)}(\zeta^{\prime }\left(-1,\frac{-j+z+1}{4z+2}\right)-\zeta^{\prime }\left(-1,\frac{-j+3z+2}{4z+2}\right)$$

$$+e^{\frac{4i\pi j}{2z+1}}\left(\zeta^{\prime }\left(-1,\frac{j+z+1}{4z+2}\right)-\zeta^{\prime }\left(-1,\frac{j+3z+2}{4z+2}\right)\right))$$

$$=(-1{)}^{z}\zeta^{\prime }\left(-1,\frac{z+1}{4z+2}\right)+\frac{1}{16z+8}(4e^{\frac{i\pi }{2z+1}}\Phi^{\prime }\left(-e^{\frac{2i\pi }{2z+1}},-1,1\right)$$

$$-8(-1{)}^{z}(2z+1)\zeta^{\prime }\left(-1,\frac{3z+2}{4z+2}\right)+i\log\left(\frac{1}{4z+2}\right)\left(\tan\left(\frac{\pi }{2z+1}\right)+i\right)\sec\left(\frac{\pi }{2z+1}\right))$$ (41)

*Proof*: Use Eq (34) and take the first partial derivative with respect to *k* and set $k=1,a=\frac{1}{1+2z}$ and simplify. Note Glaisher’s constant will be evaluated for *z* > 1. □

**Example 2.33.** *Finite sum in terms of Apéry’s constant.*


$$\sum_{j=1}^{z}(-1{)}^{j+z}e^{i\pi \left(1-\frac{2j}{2z+1}\right)}(\zeta^{\prime }\left(-2,\frac{-j+z+1}{4z+2}\right)-\zeta^{\prime }\left(-2,\frac{-j+3z+2}{4z+2}\right)$$

$$+e^{\frac{4i\pi j}{2z+1}}\left(\zeta^{\prime }\left(-2,\frac{j+z+1}{4z+2}\right)-\zeta^{\prime }\left(-2,\frac{j+3z+2}{4z+2}\right)\right))$$

$$=(-1{)}^{z}\zeta^{\prime }\left(-2,\frac{z+1}{4z+2}\right)+\frac{1}{16(2z+1{)}^{2}}(4e^{\frac{i\pi }{2z+1}}\Phi^{\prime }\left(-e^{\frac{2i\pi }{2z+1}},-2,1\right)$$

$$-16(-1{)}^{z}(2z+1{)}^{2}\zeta^{\prime }\left(-2,\frac{3z+2}{4z+2}\right)$$

$$+\log\left(\frac{1}{4z+2}\right)\tan\left(\frac{\pi }{2z+1}\right)\left(\tan\left(\frac{\pi }{2z+1}\right)+i\right)\sec\left(\frac{\pi }{2z+1}\right))$$ (42)

*Proof*: Use Eq (34) and take the first partial derivative with respect to *k* and set $k=2,a=\frac{1}{1+2z}$ and simplify. Note Apéry’s constant will be evaluated for z≥1. □

**Example 2.34.** *Finite sum in terms of Catalan’s constant.*


$$\sum_{j=1}^{z}(-1{)}^{j+z}e^{i\pi \left(1-\frac{2j}{2z+1}\right)}(\zeta^{\prime }\left(-1,\frac{-2j+2z+1}{8z+4}\right)-\zeta^{\prime }\left(-1,\frac{-2j+6z+3}{8z+4}\right)$$

$$+e^{\frac{4i\pi j}{2z+1}}\left(\zeta^{\prime }\left(-1,\frac{2j+2z+1}{8z+4}\right)-\zeta \left(-1,\frac{2j+6z+3}{8z+4}\right)\right))$$

$$=\frac{1}{8(2\pi z+\pi )}(4\pi e^{\frac{i\pi }{2z+1}}\Phi^{\prime }\left(-e^{\frac{2i\pi }{2z+1}},-1,\frac{1}{2}\right)$$

$$+4Ce^{i\pi z}(2z+1)+i\pi \log\left(\frac{1}{4z+2}\right)\tan\left(\frac{\pi }{2z+1}\right)\sec\left(\frac{\pi }{2z+1}\right))$$ (43)

*Proof*: Use Eq (34) and take the first partial derivative with respect to *k* and set k=1,a=0 and simplify. □

### 2.5 A special case when j=z=1

In this section we look at the product of quotient gamma functions previously studied by Gauss [DLMF, 5.5.5] and derived using contour integration. Products of gamma functions over certain sequences, like the ones in this section, are related to the Barnes G-function [DLMF, 5.17.1]. This function generalizes the notion of the superfactorial and has its own set of functional equations and reflection formulas.

**Example 2.35.** *A finite product involving gamma of power of 3.*


$$\prod_{p=0}^{n}\frac{\Gamma \left(\frac{1}{4}3^{-p-1}\left(3a+3^{p}\right)\right)\Gamma \left(\frac{1}{4}3^{-p-1}\left(3a+5\times 3^{p}\right)\right)\Gamma {\left(\frac{1}{4}\left(3^{-p}a+3\right)\right)}^{2}}{\sqrt{3}\Gamma \left(\frac{1}{4}3^{-p-1}\left(3a+7\times 3^{p}\right)\right)\Gamma \left(\frac{1}{4}3^{-p-1}\left(3a+11\;3^{p}\right)\right)\Gamma {\left(\frac{1}{4}\left(3^{-p}a+1\right)\right)}^{2}}$$

$$=\frac{\Gamma \left(\frac{1}{4}(3a+1)\right)\Gamma \left(\frac{1}{4}\left(3^{-n}a+3\right)\right)}{\Gamma \left(\frac{3(a+1)}{4}\right)\Gamma \left(\frac{1}{4}\left(3^{-n}a+1\right)\right)}$$ (44)

*Proof*: Use Eq (13) and set m=0,j=z=1 and simplify in terms of the Hurwitz zeta function. Next take the first partial derivative with respect to *k* and set *k* = 0 then take the exponential of both sides. □

**Example 2.36.** *An infinite product involving gamma of power of 3.*


$$\prod_{p=0}^{\infty }\frac{\Gamma \left(\frac{1}{4}3^{-p-1}\left(3a+3^{p}\right)\right)\Gamma \left(\frac{1}{4}3^{-p-1}\left(3a+5\times 3^{p}\right)\right)\Gamma {\left(\frac{1}{4}\left(3^{-p}a+3\right)\right)}^{2}}{\sqrt{3}\Gamma \left(\frac{1}{4}3^{-p-1}\left(3a+7\times 3^{p}\right)\right)\Gamma \left(\frac{1}{4}3^{-p-1}\left(3a+11\;3^{p}\right)\right)\Gamma {\left(\frac{1}{4}\left(3^{-p}a+1\right)\right)}^{2}}$$

$$=\frac{\Gamma \left(\frac{3}{4}\right)\Gamma \left(\frac{1}{4}(3a+1)\right)}{\Gamma \left(\frac{1}{4}\right)\Gamma \left(\frac{3(a+1)}{4}\right)}$$ (45)

*Proof*: Use Eq (44) and take the limit of the right-hand side as n→∞ and simplify. This form can also be derived using Gauss’ multiplication formula Eq (5.5.6) in [35]. □

## Discussion

**Fig 1:**
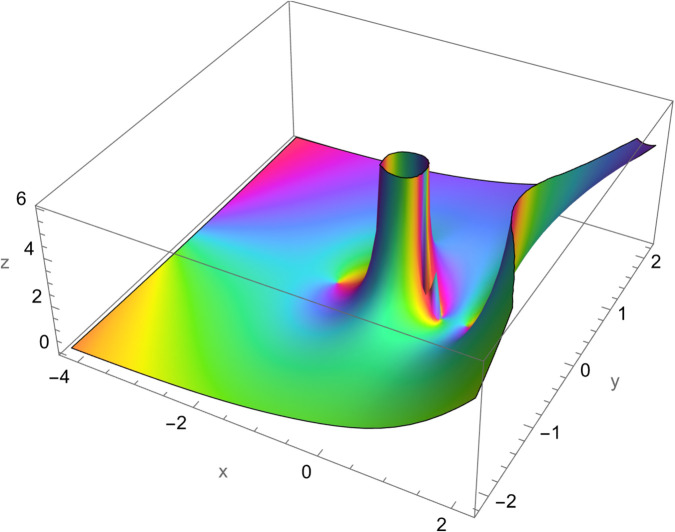
Plot of $f\;(r)=\left|\frac{\tan^{3}\left(\frac{\pi }{4}+\frac{r}{6}\right)\tan\left(\frac{1}{4}(\pi +2r)\right)}{\tan^{3}\left(\frac{\pi }{4}+\frac{1}{6r}\right)\tan\left(\frac{1}{4}\left(\pi +\frac{2}{r}\right)\right)}\right|$, r∈ℝ.

**Fig 2:**
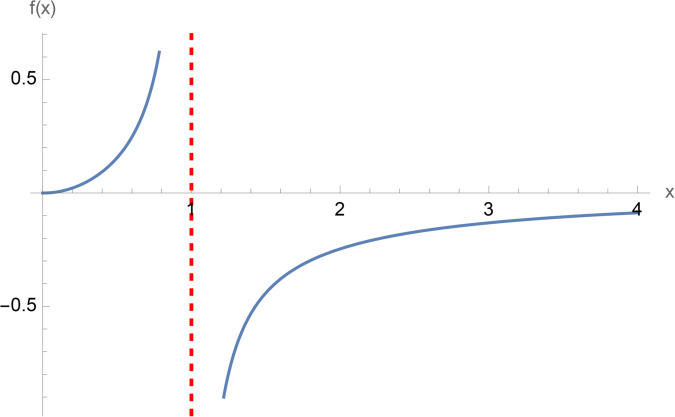
Plot of $Re\left(\frac{x^{7/3}\log\left(\log\left((-1{)}^{5/8}x\right)\right)}{1-x^{4}}\right)$, x∈ℝ.

**Fig 3:**
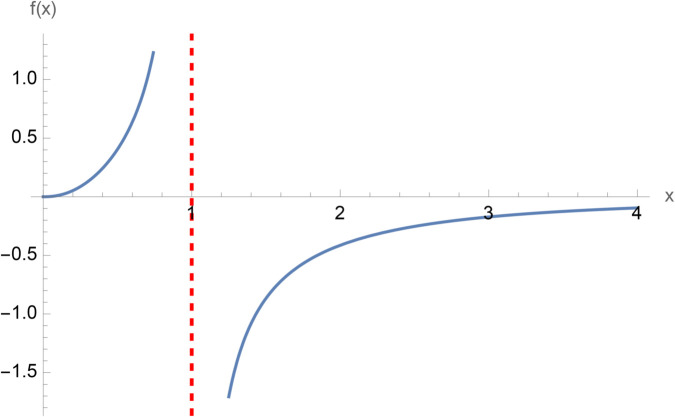
Plot of $Im\left(\frac{x^{7/3}\log\left(\log\left((-1{)}^{5/8}x\right)\right)}{1-x^{4}}\right)$, x∈ℝ.

In this study, we employed a contour integration method to establish mathematical expressions for double finite sums involving the Hurwitz-Lerch zeta function. While this approach is generally easy, its application to the double finite sum of the secant function posed difficulties during evaluation. Our challenges encompassed both the simplification of the secant function’s representation within the contour integral and the identification of specific values where the double product finite sum becomes simpler. The significance of this research lies in the deriving closed-form solutions through these methodologies. Consequently, we have introduced formulas to the existing body of knowledge, with the hope that they will prove valuable to the academic community (Figs 1–3).

## Conclusion

In this paper, we have presented a method for deriving double finite sum and products formulae involving the Hurwitz-Lerch zeta function along with some interesting special cases using both contour integration and well known algebraic techniques. We plan to apply these methods to derive other multiple sum and product formulae involving other special functions in future work.
